# Hospital doctors in Ireland working through COVID-19 pandemic: learning from individual experience: Niamh Humphries

**DOI:** 10.1093/eurpub/ckac129.044

**Published:** 2022-10-25

**Authors:** N Humphries, J-P Byrne, J Creese

**Affiliations:** 1 RCSI Graduate School of Healthcare Management, Dublin, Ireland; 2 College of Life Sciences, University of Leicester, Leicester, UK

## Abstract

**Background:**

This study was part of a 5-year, HRB-funded research project about hospital doctor retention and emigration.

**Methods:**

In 2021, we conducted a Mobile Instant Messaging Ethnography (MIME) with 28 hospital doctors in Ireland. This involved interviewing doctors via Zoom and engaging them in a 12-week work-related conversation via WhatsApp.

**Results:**

Our findings illustrate that the pandemic intensified already difficult working conditions. Respondents described working in an under-staffed and under-resourced system, in which they were unable to protect their own wellbeing or achieve a work-life balance. Morale was low and few had hope of health system improvement.

**Conclusions:**

The findings reveal a workforce under strain and raise concerns about health worker wellbeing and health worker attrition, post-pandemic. However, they also highlight the importance (and value) of listening to the voices of frontline health workers and using their insights to inform and enhance retention policies.

